# Supplementary material for: Leaf-associated microbiomes of grafted tomato plants
Source: Sci Rep. 2019 Feb 11;9:1787. doi: 10.1038/s41598-018-38344-2 (PMC6370777; doi:10.1038/s41598-018-38344-2)
Supplement: Supplementary file 1 — Supplementary Information [file 41598_2018_38344_MOESM1_ESM.pdf]

Supplementary Information:

Leaf-associated microbiomes of grafted tomato plants

Hirokazu Toju[1,2*], Koji Okayasu[3] and Michitaka Notaguchi[2,3]

[1]Center for Ecological Research, Kyoto University, Otsu, Shiga 520-2133, Japan

[2]Precursory Research for Embryonic Science and Technology (PRESTO), Japan Science and Technology Agency, Kawaguchi, Saitama 332-0012, Japan

[3]Graduate School of Bioagricultural Sciences, Nagoya University, Furo-cho, Chikusa-ku, Nagoya, Aichi, 464-8601 Japan

*Correspondence and requests for materials should be addressed to H.T. (email: toju.hirokazu.4c@kyoto-u.ac.jp).

This file includes:

Supplementary Table 1. Tomato cultivars used in this study.

Supplementary Figure 1. Relationships between the number of sequencing reads and that of OTUs.

Supplementary Table 1. Tomato cultivars used in this study.

**Stock cultivars**	**Traits/origin**	**Providing company**
Solanum lycopersicum cv Chibikko	A dwarf, fresh-eating/ornamental cultivar	Marutane Co., Ltd.
Solanum lycopersicum cv Ganbarune	A stock cultivar with increased disease resistance	Aisan Seed Co., Ltd.
Solanum lycopersicum cv M82	A processing tomato cultivar, originated from the United States of America	–
Solanum lycopersicum cv Micro-Tom	A dwarf, ornamental cultivar generated by crossing 'cv Florida Basket' and 'cv Ohio 4013-3'	–
Solanum lycopersicum cv Regina	A dwarf, ornamental cultivar, originated from Latin America	Sakata Seed Co.
Solanum lycopersicum cv Spike	A stock cultivar with enhanced growth	Aisan Seed Co., Ltd.
Solanum lycopersicum cv Triper	A stock cultivar with increased disease resistance	Aisan Seed Co., Ltd.
Solanum lycopersicum cv Momotaro-Haruka	A disease-resistant, fresh-eating cultivar	Takii Co., Ltd.

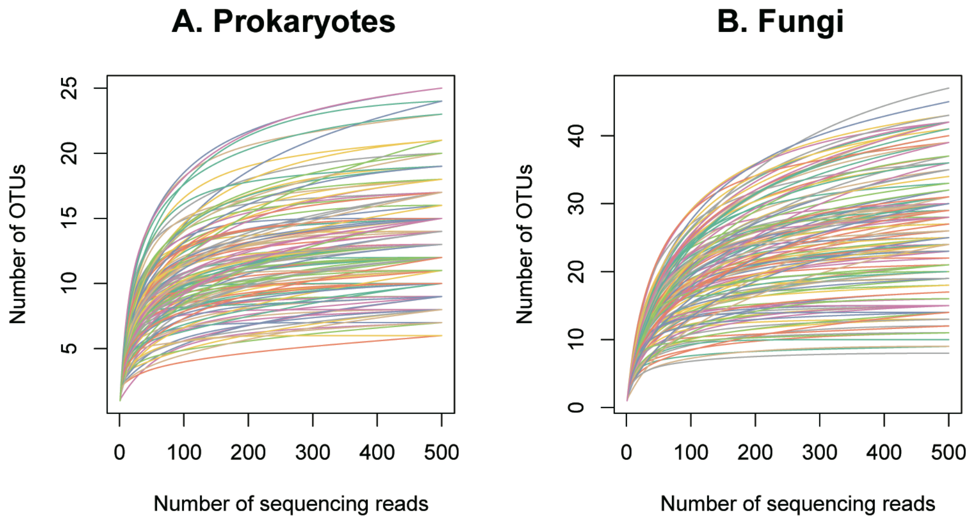

Supplementary Figure 1. Relationships between the number of sequencing reads and that of OTUs.
